# Bio-production of gaseous alkenes: ethylene, isoprene, isobutene

**DOI:** 10.1186/s13068-018-1230-9

**Published:** 2018-08-29

**Authors:** James Wilson, Sarah Gering, Jessica Pinard, Ryan Lucas, Brandon R. Briggs

**Affiliations:** Department of Biological Sciences, University of Alaska Anchorage, Anchorage, AK 99508 USA

## Abstract

To reduce emissions from petrochemical refinement, bio-production has been heralded as a way to create economically valuable compounds with fewer harmful effects. For example, gaseous alkenes are precursor molecules that can be polymerized into a variety of industrially significant compounds and have biological production pathways. Production levels, however, remain low, thus enhancing bio-production of gaseous petrochemicals for chemical precursors is critical. This review covers the metabolic pathways and production levels of the gaseous alkenes ethylene, isoprene, and isobutene. Techniques needed to drive production to higher levels are also discussed.

## Background

**Fig. 1:**
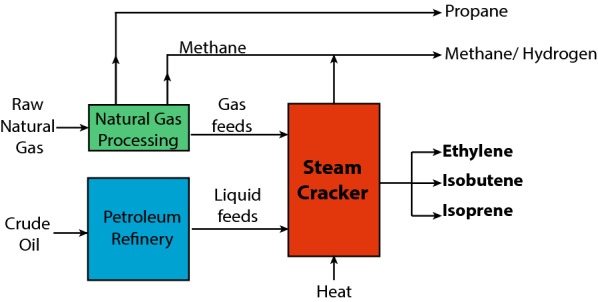
Petrochemical process of converting natural gas and crude oil into industrial building blocks using steam cracking, which is highly energy intensive

In today’s world, the impact of fossil fuels is inescapable. In addition to energy, fossil fuel products are used to create high-value industrial chemicals and polymers that permeate our society. Currently, petrochemicals are produced via steam cracking of crude petroleum products, which requires high temperature and pressure, and anoxic conditions (Fig. 1). This process is energetically demanding, requiring ~ 14% of the total energy industry [1]. In addition, the molten salt used to reduce coke formation in the refinement process leads to additional requirements for contaminant disposal [2]. Producing petrochemicals in this fashion emits massive quantities of greenhouse gases (GHG) and potential environmental contaminants that have a myriad of effects on our environment, economies, and species as a whole. One avenue of research that is potentially more carbon neutral and less polluting is the bio-production of chemicals. While switching production of petrochemicals from steam-cracking crude oil, to a renewable production method would not alleviate all emissions, it would redress one of the major sources [3].

Much of the effort to date has been in production of liquid biofuels or biodiesels [4]. Ethanol production from corn using yeast is a prime example of this process [5]. However, many industrially significant chemicals are gaseous in nature and are processed into higher order petrochemicals such as polyethylene (the thin plastic used in grocery bags and packaging film) detergents, fuel additives, anti-knocking agents for combustion engines, synthetic rubbers for the tire industry, adhesives, and perfumes (Table 1). The predominant hydrocarbons that are used as precursor molecules are gaseous alkenes—ethylene, isobutene, and isoprene. These chemicals can be produced naturally through various microbial processes; however, they are yet to readily reach the market in part due to inefficient pathways, or the need for expensive feedstocks [3]. Due to this hurdle, much of the current research in gaseous bio-products focuses on optimization of production organisms through genetic engineering and careful manipulation of growth conditions. This review will concentrate on genetic engineering of key microbial pathways, and enzymes to increase production levels of gaseous precursor alkenes (ethylene, isobutene, and isoprene). This will provide a picture of the current state of bio-production for these precursor molecules.

**Table 1:** Products produced from alkenes

***Ethylene***	
Food packaging	Stretch film
Shrink wrap	Detergents
Containers	Alcohols
Pipes	Adhesives
Garbage bags	Paints
Polyester fiber (textiles)	Paper coatings
Bottles	Industrial ethanol
Antifreeze	Surfactants
Shampoo	Personal care products
Kitchen cleaners	Construction industry
Solvents	Synthetic rubber
Fuels	
***Isobutene***	
Insecticides	Roofing material
Latex	Car lubricants
Balloons	Gasoline oxygenate
Medical devices	Fuel additive
Waterproof material	Food articles
***Isoprene***	
Synthetic rubber	Adhesives
Baby bottle nipples	Paints and coatings
Toys	Tires
Shoe soles	Elastic films

## Overview of metabolic pathways

### Ethylene

Three pathways for biological ethylene production have been identified (1)* S*-adenosyl-methionine (SAM) pathway, (2) 4-(methylsulfanyl)-2-oxobutanoate (KMBA) pathway, and (3) 2-oxoglutarate pathway [6, 7]. Plants naturally produce ethylene as a hormone that modulates growth and development using the SAM pathway. The SAM pathway is a two-step reaction that first converts SAM into 1-aminocyclopropane-1-carboxylic acid (ACC) by ACC synthase. ACC oxidase then catalyzes the release of ethylene and cyanide. Plants then detoxify the cyanide by converting it to β-cyanoalanine. Utilization of this pathway in microorganisms for biotechnological applications might require additional engineering as not all host strains contain natural cyanide mitigation pathways [8].

In addition to plants, a variety of bacteria and fungi also naturally produce ethylene using the KMBA pathway. KMBA is produced through a NADH:Fe(III)EDTA oxidoreductase-mitigated reaction with methionine. The pathway produces ethylene in trace amounts, but has been observed to be enhanced under ammonia limitations [9]. It is hypothesized that the formation of KMBA is a way to recover amino nitrogen from methionine that spontaneously leads to the formation of ethylene [9]. Only trace amounts of ethylene are produced through this pathway, thus it has not been as extensively studied.

**Fig. 2:**
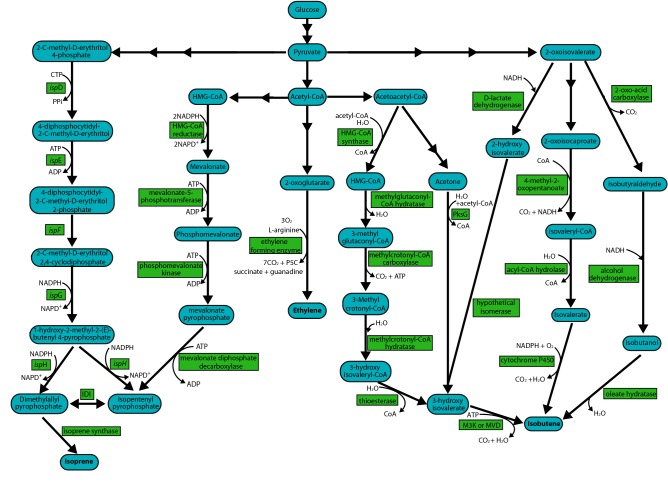
Pathways from glycolytic and citric acid cycle products to isoprene, ethylene, and isobutene. HMG-CoA, 3-hydroxy-3-methylglutaryl-CoA; P5C, l-delta 1-pyrroline-5-carboxylate; *isp*D, 4-diphosphocytidyl-2-C-methylerythritol synthetase; *isp*E, 4-diphosphocytidyl- 2-C-methyl-d-erythritol kinase; *isp*F, 2-C-methyl-d-erythritol-2,4-cyclodiphosphate synthase; *isp*G, (*E*)-4-hydroxy-3-methylbut-2-methyl-d-erythritol-2,4-cyclodiphosphate synthase; *isp*H, HMBPP reductase; IDI, IPP isomerase; PKSG, 3-hydroxy-3-methylglutaryl-ACP synthase

The 2-oxoglutarate pathway is used by several microbes within the *Pseudomonas* and *Penicillium* genus. Ethylene is produced from 2-oxoglutarate using ethylene-forming enzyme (EFE) (Reaction 1) (Fig. 2). However, stoichiometry of cell-free extracts indicates a “dual-circuit” reaction that uses 2-oxoglutarate but does not produce ethylene (Reaction 2) [10, 11]. Further work has confirmed the dual-circuit nature of this enzyme, but indicated that these two reactions can be separated because l-arginine analogs still produce ethylene without hydroxylation of the analog [11]. Additionally, X-ray crystallography of EFE from *P. syringae* in complex with manganese as well as 2-oxoglutarate has shown that l-arginine induces a conformational twist of several amino acid residues (Glu84, and Tyr192) into the active site [12, 13]. Future work on separating these two reactions may lead to more carbon-efficient production. Nevertheless, this pathway is the most biotechnologically promising pathway because it has the highest rates of ethylene production, only one additional enzyme is needed, and 2-oxoglutarate is a common substrate produced by many organisms through the tricarboxylic acid (TCA) cycle. This makes it possible to engineer into many different organisms [14, 15]. Thus, most research has focused on understanding and enhancing the 2-oxoglutarate pathway for production of ethylene.

1 $$2 {\text{-oxoglutarate}} + {\text{O}}_{2} \to {\text{ethylene}} + {\text{H}}_{2} {\text{O}} + 3 {\text{CO}}_{2}$$

2 $$ 2 \text{-Oxoglutarate} + \text{O}_{2} + \text {L-arginine} \to \text{succinate} + \text{CO}_{2} + \text{H}_{2} \text{O} + \text{guanidine} + \text{1-pyrroline-5-carboxylate}$$

### Isoprenoid pathways

The two main isoprenoid pathways that can lead to isobutene are the methylerythritol-phosphate (MEP) pathway and mevalonate (MVA) pathway (Fig. 2). Both pathways produce isoprenoid precursors of dimethylallyl diphosphate (DMAPP) and isopentenyl pyrophosphate (IPP). Most bacteria use the MEP pathway, whereas the MVA pathway is present in eukaryotes and archaea [16]. The primary feedstock molecule for the MEP pathway is pyruvate. 1-deoxy-d-xylulose-5-phosphate (DXP) is made through a condensation reaction with pyruvate and glyceraldehyde-3-phosphate. This is eventually converted to hydroxy-2-methyl-2-butenyl-4-diphosphate (HMBPP), which is the precursor for IPP and DMAPP. An IPP isomerase (IDI) catalyzes the reversible reaction of IPP to DMAPP. In this pathway, 1-deoxy-d-xylulose-5-phosphate synthase (DXS), 1-deoxy-d-xylulose-5-phosphate reductoisomerase (DXR), and IDI have been identified as the rate-limiting enzymes [17].

The MVA pathway starts with two molecules of acetyl-CoA to form 3-hydroxy-3-methylglutaryl coenzyme A (HMG-CoA). The rate-limiting step in this pathway is the reduction of HMG-CoA to mevalonate by the HMG-CoA reductase [18]. Mevalonate is then phosphorylated twice to form mevalonate-5-diphosphate. This is decarboxylated by mevalonate-5-diphosphate decarboxylase (MVD) to form IPP. Isoprene can be produced from either pathway from DMAPP using isoprene synthase (ISPS). The MEP pathway is energetically balanced and theoretically more carbon efficient than the MVA pathway (30.2% vs. 25.2% mass yield on glucose) [16]. However, the MVA pathway is well characterized and is amenable to metabolic pathway engineering [19]. Furthermore, current engineering efforts have had higher success using the MVA pathway [20–22].

### Isobutene pathways

Isobutene can be produced from three different intermediates—isobutanol, isovalerate, and 3-hydroxyisovalerate (Fig. 2). There are a few natural producers of isobutanol such as *Saccharomyces cerevisiae* and *Lactococcus lactis* [23]. There are also engineered production strains of *E. coli* that produce isobutanol via acetolactate and 2-oxoisovalerate [24] (Fig. 2). The dehydration of isobutanol to isobutene is catalyzed by an oleate hydratase [25]. The reaction mechanism for this has not been elucidated, but the natural reaction of an oleate hydratase is the conversion of oleic acid to (*R*)-10-hydroxystearate with narrow substrate specificity. A patent by Marliere searched 165 homologs of oleate hydratase and found several that can produce isobutene [25]. No information has been given about the reaction kinetics or production rates.

Natural production of isobutene was first mentioned by Fukuda et al. [26]. Of the 178 tested organisms, 33 fungi, 31 yeasts, and 6 bacteria produced trace amounts of isobutene. The highest natural production value was found in the yeast *Rhodotorula minuta* with a rate of 0.45 mg L^−1^ h^−1^ and 41 μg g^−1^ h^−1^ [27]. It was later found that *R. minuta* produces isobutene by the decarboxylation of isovalerate using a microsomal cytochrome P450 (Fig. 2). The pathway from glucose to 2-oxoisocaproate is generally used for leucine biosynthesis. In the subsequent steps, two CO_2_ equivalents are removed. This pathway requires 2 mol of pyruvate and 2 mol of acetyl-CoA, which gives a low theoretical maximum yield of isobutene per mol of glucose. Furthermore, the cytochrome P450 requires a heme moiety, which is not well suited for recombinant expression in bacteria.

Isobutene production via 3-hydroxyisovalerate is a derivative of the MVA pathway. HMG-CoA is produced through the MVA pathway but instead of reducing it to methionine, HMG-CoA is dehydrated to 3-methylglutaconyl-CoA, which is subsequently converted to 3-hydroxyisovalerate. The initial steps in the MVA pathway require acetyl-CoA and acetoacetyl-CoA for the production HMG-CoA. This leads to a requirement of 3 mol of pyruvate for 1 mol of isobutene. There is a patent that produces 3-hydroxyisovalerate from acetyl-CoA and acetone, but that still has the same requirement of 3 mol of pyruvate per mol of isobutene [28]. The last enzyme in this pathway is MVD, which has the ability to decarboxylate 3-hydroxyisovalerate (3-HIV) to isobutene as a side reaction (Reaction 3). Recently, an enzyme in the MVA pathway of *Picrophilus torridus* has been identified as a mevalonate-3-kinase (M3K) [29]. This newly discovered enzyme has the highest recorded rate of isobutene formation (507 pmol min^−1^ g cells^−1^) and acts through catalyzing the phosphorylation of 3-HIV into an unstable 3-phosphate intermediate that undergoes spontaneous decarboxylation to form isobutene (Reaction 4).

3 $${\text{C}}_{5} {\text{H}}_{9} {\text{O}}_{3} + {\text{ATP}} \to {\text{C}}_{4} {\text{H}}_{8} + {\text{CO}}_{2} + {\text{P}}_{\text{i}}$$

4 $${\text{C}}_{5} {\text{H}}_{9} {\text{O}}_{3} + {\text{ATP}} \to {\text{C}}_{5} {\text{H}}_{8} {\text{O}}_{3} {\text{P}} \to {\text{C}}_{4} {\text{H}}_{8} + {\text{CO}}_{2} + {\text{P}}_{\text{i}}$$

## Genetic engineering

### Ethylene

Only one enzyme is needed for ethylene production from common metabolites. As such, depending on the organism heterologous expression of the *efe* gene can produce ethylene from various forms of carbon (Table 2). For example, ethylene was synthesized from carbon dioxide when *efe* was expressed in *Synechocystis* [30–32]. Additionally, ethylene is produced from cellulose material or corn stover and manure when the *efe* gene was engineered into *Trichoderma reesei* or *Escherichia coli,* respectively [33–35]. Heterologous expression of the *efe* gene has also occurred in *Azotobacter vinelandii, Rhodospirillum rubrum, Pseudomonas syringae*, and *Saccharomyces cerevisiae* [36–39] (Table 2).

**Table 2:** Metabolic engineering of microorganisms for ethylene production

Organism	Description	Production	References
*Escherichia coli* MG1655	*efe* cloned into a high-copy plasmid downstream of a *lac* inducible promoter	4.39e^−5^ mL^−1^ A_600_^−1^ mL^−1^ (peak production)	[3]
*Escherichia coli* JM109	Transformed with *efe* from *P. syringae* on pUC19 backbone	0.23 mL L^−1^ h^−1^	[34]
*Escherichia coli* JM109	Overexpressed EFE from *P. syringae*	10 mL h^−1^ g^−1^ dcw	[86]
*Synechocystis* sp. PCC 6803	Overexpressed EFE from *P. syringae* and blocked TCA intermediate enzymes	9.7 mL L^−1^ h^−1^	[32]
*Synechocystis* sp. PCC 6803	Genome integration of *efe*	0.44 mL L^−1^ h^−1^	[87]
*Synechocystis* sp. PCC 6803	4 copies of *efe*, modified TCA cycle, and partial deletion of *ntcA*	2.64 mL L^−1^ h^−1^ A_730_^−1^	[42]
*Synechocystis* sp. PCC 6803	Chromosomal insertion of codon-optimized *efe* from *P. syringae* under pea *pshA* promoter	5.65 mL L^−1^ h^−1^	[30]
*Synechocystis* sp. PCC 6803	Integrated *efe* cassette with modified ribosomal binding site	0.72 mL L^−1^ h^−1^ A_730_^−1^	[31]
*Synechocystis* sp. PCC 6803	Codon-optimized *efe* expressed from pVZ321 with *lacI* repressor, *Trc* promoter, pol-linker, and transcription terminators from pTrc99a	0.2 mL L^−1^ h^−1^	[15]
*Saccharomyces cerevisiae*	Multicopy plasmid expression of oxidase *Aox1* and *nox*	0.23 mL L^−1^ h^−1^	[88]
*Saccharomyces cerevisiae*	*efe* cloned into pYX212-EFE under TPI1 promoter	0.19 mL L^−1^ h^−1^	[89]
*Saccharomyces cerevisiae*	Expressed *P. syringae efe*	0.34 mL L^−1^ h^−1^	[39]
*Synechococcus* sp. PCC 7942	*efe* cloned in pUC303 with promoter and terminator from *psbAI*	0.03 mL L^−1^ h^−1^ A_730_^−1^	[90]
*Synechococcus elongatus* PCC 7942	*P. syringae efe* was inserted at the *psbAI* locus	0.451 mL L^−1^ h^−1^ A_730_^−1^	[91]
*Trichoderma viride*	Integrated *efe* from *P. syringae* into chromosome under the control of *cbh1* promoter	0.00106 mL h^−1^ g^−1^ dcw	[92]
*Trichoderma reesei*	Integrated into chromosome 4 *efe* genes under *pgkL* promoter	0.0041 mL L^−1^ h^−1^	[33]
*Pseudomonas syringae*	Cloned *efe* into a high-copy plasmid pMEFE1	8 mL L^−1^ h^−1^ A_610_^−1^	[93]
*Pseudomonas putida* KT2440	Integrated *efe* from *P. syringae* at five 16S rDNA sites	64.04 mL h^−1^ g^−1^ dcw	[41]

Efforts to produce a stable and efficient ethylene producing strain have included protein engineering of EFE, increasing the copy number of the *efe* gene, altering promoter control, modifying ribosomal binding sites, and modifying metabolic pathways (Table 2). Protein engineering of EFE has included codon optimization for the host and site-directed mutagenesis [30, 32, 36]. These efforts along with homology modeling and X-ray crystallography have identified key amino acids in EFE required for ethylene production [13, 36]. This paves the way for directed protein engineering to further enhance ethylene production [40].

In *Pseudomonas putida*, production of ethylene reached 64 mL h^−1^ g^−1^ of dry weight when EFE was expressed from 5 of the 7 16S rRNA sites [41]. One of the higher producing strains of *Synechocystis* has been engineered to express EFE under the PcpcB promoter. The TCA cycle was also modified by blocking 2-oxoglutarate decarboxylase and succinic semialdehyde dehydrogenase, EFE was overexpressed, and 2-oxoglutarate permease from *E. coli* was introduced to increase the supply of 2-oxoglutarate [32]. The production of this strain was further enhanced by a partial deletion of the transcription factor nitrogen control A (NtcA), and four copies of the *efe gene* were introduced [42]. The peak production rate for this strain of *Synechocystis* was pushed to 2.463 mL L^−1^ h^−1^ A_730_^−1^. Transgenic expression of a fused soybean ACC synthase with a tomato ACC oxidase in *E. coli* was used to isolate and purify a novel fusion protein for ethylene production. Using this partially purified protein with *S*-adenosyl-l-methionine as substrate, researchers demonstrated a production rate of 6.0 nmol h^−1^ mg^−1^ [43].

### Isoprene

A variety of organisms naturally produce isoprene, such as humans, plants, yeast, and bacteria [44, 45]. So far, the best microbial producer of isoprene was found to be *Bacillus subtilis* with a production rate of 12.78 nmol g^−1^ h^−1^ [44]. However, the enzymatic pathway for this production has yet to be determined. Of all the organisms, plants produce the highest amount of isoprene, an estimated 600 million tonnes per year [46]. Isoprene is synthesized in the chloroplast in a process that seems to be induced by heat stress [47]. Unfortunately, it is difficult to harvest from leafy canopies. Nevertheless, plant enzymes that produce isoprene have been determined and represent a source of exogenous pathways to engineer into microbes for production [16].

Isoprene synthase has been characterized in several plants, mainly in the rosids clade such as *Populus alba* (poplar), *Quercus petraea* (oak), *Eucalyptus globulus* (eucalyptus), *Salix discolor* (willow), and *Pueraria montana* (kudzu vine) [48–50]. Biochemical studies of these ISPS proteins have shown that these enzymes are strongly temperature dependent with an optimal temperature of 40–50 °C and have high Michaelis constants (*K*_m_) [49, 51, 52]. The *K*_m_ values from DMAPP range from 18.3 to 0.03 mM with *Eucalyptus globulus* having the lowest value [16]. This requires high-substrate (DMAPP) concentrations for the ISPS to perform and is not biotechnologically advantageous. Therefore, genetic engineering studies have attempted to enhance the ISPS performance (Table 3). For example, researchers have codon-optimized *ispS* for expression in *Synechocystis* sp. PCC6803 [53], removed the chloroplast targeting peptide [48, 54], site mutagenesis [55], and performed directed evolution [56]. Additionally, to overcome the low substrate affinity of ISPS for DMAPP, a fusion protein of overexpression of *S. cerevisiae* IDI and *P. alba* ISPS was generated. Mutants expressing this fusion protein were shown to have higher production, up to 4.6 mg h^−1^ L^−1^, of isoprene than in mutant strains overexpression either of the two enzymes [57]. A recent study has suggested that there may be other more favorable ISPS proteins for biotechnological advancement [58]. Ilmen et al. found a novel *ispS* gene in the *Ipomoea batata*, a member of the asterids clade. Compared to the most similar ISPS protein (*Quercus petraea*), the *I. batata* ISPS protein is only 55% similar. Ilimen et al. went further and compared the isoprene production rates of ISPS proteins from different plants that were cloned into *E. coli*, thus allowing for a direct comparison of the unmodified proteins. The highest isoprene production rate was 40 μg L^−1^ h^−1^ by the *I. batata* ISPS. This was two times more than the second highest production rate produced by the poplar ISPS. Other studies that have expressed ISPS from kudzu and poplar in *E. coli* without further optimization found slightly higher production values than found in Ilimen et al. but the highest was 33 μg L^−1^ h^−1^ [22, 59]. While the *K*_m_ of the *I. batata* ISPS has not been determined, the faster rates of isoprene production warrant further investigation. Furthermore, a comparative analysis may identify further residues for site mutagenesis.

**Table 3:** Metabolic engineering of microorganisms for isoprene production

Organism	Description	Productivity	Yield (mg g^−1^ dcw)	Titer (g L^−1^)	Reference
*Escherichia coli* BL21	*ispS* from *I. batatas*	40 µg L^−1^ h^−1^			[58]
*Escherichia coli* BL21	Engineered with *P. alba ispS* and *S. cerevisiae* MVA pathway	11,083 µg L^−1^ h^−1^		0.532	[94]
*Escherichia coli* BL21	DXS, DXR, and IDI from *S. pneumoniae* were overexpressed, *ispA* was weakened; *P. alba ispS*	829 µg L^−1^ h^−1^		0.0199	[95]
*Escherichia coli* BL21	Two component system (1) *E. coli* optimized for mevalonate production from sugar was used as a feedstock for (2) *E. coli* engineered with MVA and *ispS*	230,000 µg L^−1^ h^−1^		11.0	[96]
*Escherichia coli* BL21	*ispS* from *P. alba*, +*mvaE, mvaS* from *E. faecalis*, +*mvk*, PMK, MVD, IDI from *S. cervisiae*, + *mvk* from *M. mazei, *+* pgl* from *E. coli*	2,000,000 µg L^−1^ h^−1^	850	60	[97]
*Escherichia coli* BL21	*ispS* from *P. nigra,* +DXS, DXR from *E. coli*			0.16	[59]
*Escherichia coli* BL21	*ispS* from *P. nigra,* +DXS, DXR from *B. subtilis*			0.31	[59]
*Escherichia coli* BL21	*ispS* from *P. montana*, + DXS*, ispG, ispH, ipi, ispE,* DXR*, ispD, ispF* from *E. coli*, selection of translation initiation regions and adjustment of gene order in the superoperon	277 µg L^−1^ h^−1^		0.005	[22]
*Escherichia coli* BL21	*ispS* from *P. montana*, + *hmgS, hmgR* from *E. faecalis*, + *atoB* from *E. coli*, +*fni, mk, pmk, pmd* from *S. pneumoniae*, selection of translation initiation regions	17,778 µg L^−1^ h^−1^		0.32	[22]
*Escherichia coli* BL21	2 *mvaE, mvaS* from *E. faecalis*, + ERG12, ERG8, ERG19, IDI from *S. cerevisiae*, codon-optimized *ispS* from *P. alba*, *mvaS* gene mutation			6.3	[98]
*Escherichia coli* BL21	Codon-adapted *ispS* from *P. alba*, +DXS, DXR, IDI from *E. coli*, adjustment of gene order in the polycistron	2727 µg g^−1^ dcw h^−1^			[17]
*Escherichia coli* BL21	Truncated ispS from *P. alba*, +DXS, DXR, IDI, *pgl, fldA, ispG* from *E. coli*, +*ispH* from Anabaena, +*ispG* system from *T. elongatus*			8.4	[99]
*Escherichia coli* BL21	Enhanced MEP pathway and combined with MVA pathway	52,500 µg L^−1^ h^−1^			[100]
*Escherichia coli* MG1655	Codon-optimized *ispS* from *P. trichocarpa*, augmented MVA pathway, and deleted genes involved in aceto-CoA byproduct formation			1.832	[101]
*Synechococcus elongatus*	Fused ISPS from *P. alba* with IDI from *S. cerevisiae*	4600 µg L^−1^ h^−1^			[57]
*Synechocystis* sp. PCC 6803	Fused ISPS with CPCB (phyocyanin) to increase production		5.4		[102]
*Synechocystis* sp. PCC 6803	codon-optimized *ispS* from *P. montana*	2.08 µg g^−1^ dcw h^−1^			[103]
*Synechocystis* sp. PCC 6803	Codon-optimized *ispS* from *P. montana*		0.12	3.2	[104]
*Synechocystis* sp. PCC 6803	Engineered psbA2 promoter-driven *ispS* and codon optimized from *P. montana*	40 µg L^−1^ h^−1^			[104]
*Synechocystis* sp. PCC 6803	Codon-optimized *P. montana ispS*	2 µg L^−1^ h^−1^			[105]
*Synechocystis* sp. PCC 6803	Codon-optimized *P. montana ispS*	63 µg L^−1^ h^−1^			[20]
*Synechocystis* sp. PCC 6803	Codon-optimized kudzu *ispS* under *rbcL* promoter	1.16 µg L^−1^ h^−1^ A_750_^−1^			[53]
*Bacillus subtilis*	Engineered DXS, DXR			1e^−6^	[106]
*Saccharomyces cerevisiae*	Multiple copies of codon-optimized *ispS* from *P. montana*	7 µg L^−1^ h^−1^		5e^−4^	[107]
*Saccharomyces cerevisiae*	2 copies of codon-optimized *ispS* from *P. alba*, +tHMG1, IDI, ACS2, ERG10 from *S. cerevisiae*, down-regulation of ERG20 by promoter replacement		25	0.037	[108]
*Saccharomyces cerevisiae*	Enhanced Gal4p supply and directed evolution of *IspS*	51,388 µg L^−1^ h^−1^		3.7	[56]

In addition to identifying alternative isoprene synthases, research has been performed to optimize pathways that produce isoprene. For example, pathways that produce DMAPP can be optimized through gene overexpression, different promoters, and alternate control methods [56, 60, 61]. One recent study by Wang et al. used *S. cerevisiae,* that was previously engineered to have increased precursor supply, performed a combinatorial approach that included overexpression and deletion of competing promoters, increased the transcriptional activator Gal4p, performed directed evolution of *ispS*, while growing in a fed-batch fermentation system with dextrose as the carbon source [56]. The approach taken by Wang et al. increased the production values to 1.23 g L^−1^ h^−1^, which is the highest reported values thus far. However, production levels could be pushed with continued combinatorial approaches that further removed competing pathways or in new organisms that have higher efficiencies. For example, a methanogen has been genetically engineered to produce isoprene increasing efficiency by alleviating the need for oxygen [62]. Identification of isoprene synthase enzymes in bacteria or archaea and the natural pathways in these organisms would reduce the reliance on exogenous plant enzymes. Furthermore, since it is postulated that isoprene deals with thermal stress in plants it may be beneficial to look for these novel enzymes in thermophiles.

### Isobutene

There have only been a few attempts at genetic engineering to increase isobutene production and these have focused on the MVD protein and more recently the M3K protein. Both the MVD and M3K genes have been independently engineered into *E. coli* using plasmid-based systems [29, 63]. Gogerty et al. engineered the MVD from *S. cerevisiae* into *E. coli*. When they used error prone PCR on the MVD, one of the variants increased the production rates by 38-fold (Table 4). Rossoni et al. engineered the M3K gene into *E. coli*. Using whole cells grown in LB media expressing M3K under IPTG expression, isobutene production rates reached up to 507 pmol min^−1^ g cells^−1^ in *E. coli* [29]. It has been proposed that engineering an organism to express both M3K and MVD might raise titers, since they have alternative modes of action on 3-HIV [29]. In cell-free extracts with the M3K enzyme isobutene production reached 34 mg L^−1^ h^−1^ [29]. To be considered economically viable, the production rate of isobutene from a biological source must be around 2–4 g L^−1^ h^−1^ [63]. There is still much improvement needed before the bio-production of isobutene can be considered economically significant.

**Table 4:** Metabolic engineering of microorganisms for isobutene production

Organism	Description	Fermentation type	Productivity (pmol min^−1^ g^−1^ cells)	References
*Escherichia coli* BL21	Engineered M3K from *Picrophilus torridus*	Sealed vials	507	[29]
*Escherichia coli* BL21	Engineered MVD from *S. cerevisiae*	Sealed vials	2.5	[63]
*Escherichia coli* BL21	Variant of MVD using error prone PCR	Sealed vials	98.1	[63]

## Future directions

Significant hurdles remain in the bio-production of these gaseous alkenes to become a consistently viable alternative to current refinement methods. Development of production strains has included (1) protein engineering or directed evolution, (2) pathway construction, and (3) regulation of key enzymes or transcription factors. Each of these have helped to enhance production values, but, in general, it is the combination of multiple steps that have seen the greatest advancement in production values. Moving forward, bioinformatics will be integral to combining each of these steps to enhance production levels [64]. Advances in sequencing technology have allowed researchers to uncover more about novel enzymes and pathways [65]. Pipelines are also being produced to model metabolic networks that allows for a flux analysis and can be interrogated to determine ways to increase efficiency before the much more time-consuming steps of genetic engineering [66–68]. For example, programs and algorithms such as Cytoscape [69], k-Opt-Force [70], or Ecocyc [71] have been developed to help visualize changes in cellular flux networks. These programs take annotated genomes and model biochemical reactions with systems of differential equations. These are transformed to systems of linear equations and solved, generating vectors in a cone representing metabolic flux. Adding in transcriptomic data with changes in gene expression will then shift the flux through reactions, changing the output, allowing researchers to see where the cell directs its energy and carbon [72, 73]. Visually processing effects of changes in gene regulation allow for simplifying of metabolic engineering efforts. Techniques like these are what will provide the next set of enzymatic tools for genetic engineers to implement to push biofuel production to higher levels.

A variety of organisms have been used for bio-production of gaseous alkenes [74–76], but many display issues due to inefficient pathways or when scaling up to industrial levels. One organism is not ideal for producing the many petrochemicals needed from varying feedstocks, but there are a suite of characteristics that should be intrinsic to any organism under consideration for use as a cell factory. Ideal characteristics would be rapid growth rate, the ability to consume wide varieties of carbon sources, non-pathogenic, carbon and energy efficient pathways, and tolerance to wide ranges of temperature, pH, and solvent concentrations [77]. *E. coli* has been the typical model organism for ethylene, isoprene, and isobutene production [3, 78]. In part, because there are attenuated strains, it replicates rapidly, and its genome is well mapped and thus well suited for engineering [79]. However, *E. coli* is not appropriate for all feedstocks and the majority of microbes are uncultured or ‘biological dark matter’ and may hold clues for faster and more efficient production strains. As our knowledge of microbial genomes grows incorporating more of the typically “non-model” organisms will become more feasible. In addition, if these non-model organisms are extremophiles, it opens up the gate for wider industrial conditions to be used.

Not all hurdles for bio-production are biological. One of the hurdles in bringing biofuels to market is effectively scaling up the process from benchtop, to industrial scale bioreactors. This is due in large part to the large differences in reaction conditions common for benchtop biogas production monitoring vs industrial production, i.e., sealed headspace vessels vs. bioreactors. To our knowledge, there have not been any reports on attempts to scale-up production for any of these gaseous alkenes. It is outside the scope of this review to detail all the types of bioreactors and the modes of operation, for more detailed information see [80–82]; however, some extra considerations are discussed that must be taken regarding recovery of gaseous end products compared to liquid-phase products. The primary method for gas harvesting from bioreactors is headspace capture [29, 83], followed by either pressure concentration and/or membrane adsorption [84]. This simplifies the process of product harvesting and mitigates problems from feedback inhibition compared to liquid biofuel processes. However, safety issues with flammability must be considered when the alkenes are concentrated to high enough concentrations. Many of the pathways to produce the gaseous alkenes are aerobic and require sparging fresh air through the reactor media to replenish the collected headspace or through oxygenic microbes. For example, oxygen can be supplied by using bioreactors growing a consortium of algae and bacteria. Combinations of algae and bacteria have been used to treat wastewater with the algae providing the dissolved oxygen requirements for aerobic digestion and nitrification processes carried out by the bacteria [85]. Engineers working with biologist will be needed to design optimal bioreactors.

A multifaceted approach is needed to overcome these barriers. The ability to rapidly create optimized production strains will require further genetic engineering as well as advances in bioreactor design, bioinformatics pipelines, and identification of novel microbes and enzymes combined with the ability to accurately integrate novel pathways into both model and non-model organisms. Optimization of every stage of the process must occur, which will require multidisciplinary teams. For example, teams of engineers, modelers, and synthetic biologists can work together to design a complete optimized system from the bioreactor, to streamlining genetic engineering through deriving mathematical models of metabolic networks to predict energetic favorability [73]. Powerful new tools such as these will help drive gaseous bio-products to higher levels.
